# A new species of* Scutellaria* (Scutellarioideae, Lamiaceae) from Sichuan Province in southwest China

**DOI:** 10.7717/peerj.3624

**Published:** 2017-08-08

**Authors:** Fei Zhao, En-De Liu, Hua Peng, Chun-Lei Xiang

**Affiliations:** 1 Key Laboratory for Plant Diversity and Biogeography of East Asia, Kunming Institute of Botany, Chinese Academy of Sciences, Kunming, Yunnan, PR China; 2 University of Chinese Academy of Sciences, Beijing, PR China

**Keywords:** China, Endemisim, Morphology, *Scutellaria*, SEM observation, Taxonomy

## Abstract

*Scutellaria wuana*, a new species discovered from a xeric valley in Muli County of Sichuan Province in southwest China, is described and illustrated. Morphologically, the new species shares similarities with *S. mairei*, but can be readily distinguished by a suite of morphological characters including a white-pubescent erect stem, conspicuous leaf petioles, and a yellow corolla with a trapeziform lower-middle lip lobe. The habitat and distribution of *S. wuana* are also distinctive. The position of the new species within* Scutellaria* is examined in a phylogenetic context using the nuclear ribosomal internal and external transcribed spacers. Additionally, we examine leaf epidermal and pollen grain micromorphology of the new species and putative relatives.

## Introduction

The subfamily Scutellarioideae, as presently circumscribed, consists of five genera and about 380 species: *Holmskioldia* Retz., *Renschia* Vatke, *Scutellaria* L., *Tinnea* Kotschy ex Hook. f., and *Wenchengia* C.Y. Wu & S. Chow (Harley et al., 2004; Li et al., 2012; Li et al., 2016). With approximately 360 species (Paton, Suddee & Bongcheewin, 2016), *Scutellaria* is one of the largest genera within Lamiaceae. Although *Scutellaria* is primarily distributed in temperate regions and on tropical mountains (Paton, 1990a; Paton, 1990b), the genus can be found in most regions of the world except the Amazon basin, lowland tropical Africa, and Pacific islands (Paton, Suddee & Bongcheewin, 2016). China is perhaps the center of diversity of *Scutellaria*, with 101 species and 25 varieties reported (Li & Hedge, 1994; Zhou & Guo, 2001; Xiao & Wu, 2003; Hsieh, 2013; Xiang, 2016). Forty-six species and 11 varieties occur in southwest China (Wu & Li, 1977), with 10 taxa endemic to Sichuan (Li, 1992).

*Scutellaria* species are annual or perennial herbs or subshrubs that have various growth forms and habitats (Epling, 1942). The stems are generally quadrangular and covered with glandular or non-glandular hairs. Leaves are typically simple and polytropic in shape, but taxa within sect. *Lupulinaria* possess pinnatifid leaves. Flowers are always solitary in the axils of +/−diminished leaves or bracts. The calyx is 2-lipped, and the posterior lip is usually folded to produce a scutellum, which is a unique character of the genus. Based on inflorescence and bract characters, Paton (1990a) divided the genus into two subgenera: subgen. *Scutellaria* Briquet and subgen. *Apeltanthus* (Nevski ex Juz.) Juz. The former is characterized by having a one-sided inflorescence and flowers subtended by leaves or leaf-like bracts, while the latter has a four-sided inflorescence, with decussate flowers subtended by cucullate bracts. The subgenus *Scutellaria* was further divided into five sections (i.e., sect. *Scutellaria*, sect. *Anaspis* (Rech.f.) Paton, sect. *Perilomia* (Kunth) Epling, sect. *Salazaria* (Torrey) Paton, and sect. *Salviifoliae* (Boiss.) J.R.Edm.), and the subgenus *Apeltanthus* consists of two sections, i.e., sect. *Apeltanthus* and sect. *Lupulinaria* A. Hamilton. This infrageneric classification proposed by Paton (1990a) is the most comprehensive taxonomic treatment of the genus *Scutellaria*, but only 13 species from China were included. Thus, it is not possible to employ his divisions for the classification of the genus in China. In the updated edition of Lamiaceae in *Flora of China*, Li & Hedge (1994) divided *Scutellaria* (98 spp.) into seven groups on the basis of inflorescence, bract, calyx, and nutlet morphology.

During field trips to Sichuan Province in southwest China in July of 2011 and August of 2015, we encountered a noteworthy *Scutellaria* population in Muli County. However, we were unable to key our collections according to the descriptions from *Flora Sichuanica* (Li, 1992), *Flora Reipublicae Popularis Sinicae* (Wu & Li, 1977) and *Flora of China* (Li & Hedge, 1994). After carefully examining the specimens, we concluded that this population represents a new species of *Scutellaria* and henceforth describe and illustrate the new species.

## Materials & Methods

The morphological study is based on our field collections and herbarium specimens. Additionally, protologues of all published names in the genus *Scutellaria* from China and adjacent areasas well as related taxonomic literature (Wu & Li, 1977; Li & Hedge, 1994; Zhou & Guo, 2001; Xiao & Wu, 2003; Hsieh, 2013; Paton, Suddee & Bongcheewin, 2016) were assembled and reviewed. To verify morphological differences among the potential new species and other morphologically similar congeners, herbarium material (including types) from CDBI, E, IBK, IBSC, K, KUN, L, PE, SZ were examined (acronyms follow Thiers, 2016). After careful investigation of specimens, we deemed the potential new species to be most similar to *S. mairei* H. Lév., which guided us in further morphological comparisons.

Leaf materials for scanning electron microscopy (SEM) and light microcopy (LM) were taken from our collections (*C.L. Xiang 1200* and/or *E.D. Liu et al. 2902*) of the new species and herbarium specimens of *S. mairei* H. Lév. *(M.Fr. Ducloux 6438*). Methods for examining leaf epidermal microfeatures of both the new and closely related species followed Xiang et al. (2010). Size measurements of 25 pollen grains were made under a Leica DM2500 light microscope (Leica Microsystems GmbH, Wetzlar, Germany). The pollen samples were prepared for the SEM as described by Xiang et al. (2013).

**Table 1:** Voucher information for phylogenetic analyses and GenBank accession numbers.

Taxon	Voucher/Herbarium barcode	Location	Genbank accession number	
		ITS	ETS
*Scutellaria discolor*	Xiang CL et al., 438 (KUN)	Yunnan, China	MF193504	MF193550
*Scutellaria hainanensis*	Jiang L et al., 398 (KUN)	Hainan, China	MF193505	MF193551
*Scutellaria yunnanensis* var. *yunnanensis*	Liu Ed et al., 3037 (KUN)	Yunnan, China	MF193506	MF193552
*Scutellaria yunnanensis* var. *cuneata*	Xiang CL et al., 574 (KUN)	Yunnan, China	MF193507	MF193553
*Scutellaria obtusifolia*	Chen YP et al., EM202 (KUN)	Sichuan, China	MF193508	MF193554
*Scutellaria sichourensis*	Xiang CL et al., 566 (KUN)	Yunnan, China	MF193509	MF193555
*Scutellaria wenshanensis*	Zhao F et al., 008 (KUN)	Yunnan, China	MF193510	MF193556
*Scutellaria yangbiense*	Liu ED et al., 2238 (KUN)	Yunnan, China	MF193511	MF193557
*Scutellaria calcarata*	Shui YM et al., Z-03343396 (KUN)	Yunnan, China	MF193512	MF193558
*Scutellaria indica*	Peng H, s.n (KUN)	Hongkong, China	MF193513	MF193559
*Scutellaria indica* fo. *parvifolia*	Anonymous,554 (KUN)	Shimoda, Japan	MF193514	MF193560
*Scutellaria taiwanensis*	Liao PC, s.n. (KUN)	Taiwan, China	MF193515	MF193561
*Scutellaria mairei*	Shui YM et al., 66205 (KUN)	Yunnan, China	MF193516	MF193562
*Scutellaria tenax*	Peng H et al., 2012-017 (KUN)	Guizhou, China	MF193517	MF193563
*Scutellaria tapintzeensis* _1	Cai J et al., 15cs11358 (KUN)	Yunnan, China	MF193518	MF193564
*Scutellaria tapintzeensis* _2	Cai J et al., 15cs11371 (KUN)	Yunnan, China	MF193519	MF193565
*Scutellaria teniana*	Xiang CL et al., 288 (KUN)	Yunnan, China	MF193520	MF193566
*Scutellaria wuana* sp. nov.	Xiang CL et al., 1200 (KUN)	Sichuan, China	MF193521	MF193567
*Scutellaria tenera*	Chen YP et al., EM187 (KUN)	Jiangxi, China	MF193522	MF193568
*Scutellaria macrodonta*	Zhao F et al., 2015-006 (KUN)	Beijing, China	MF193523	MF193569
*Scutellaria likiangensis*	Xiang CL et al., 373 (KUN)	Yunnan, China	MF193524	MF193570
*Scutellaria baicalensis*	Li DZ et al., 0513 (KUN)	Liaoning, China	MF193525	MF193571
*Scutellaria viscidula*	Zhao F, 2015-009 (KUN)	Hebei, China	MF193526	MF193572
*Scutellaria orthocalyx*	Xiang CL, 185 (KUN)	Yunnan, China	MF193527	MF193573
*Scutellaria subintegra*	Chen YP, EM223 (KUN)	Guangxi, China	MF193528	MF193574
*Scutellaria axilliflora*	Hu GX, H144 (KUN)	Fujian, China	MF193529	MF193575
*Scutellaria shweliensis*	Zhao F et al., ZF0068 (KUN)	Yunnan, China	MF193530	MF193576
*Scutellaria hunanensis*	Hu GX, H96 (KUN)	Hunan, China	MF193531	MF193577
*Scutellaria franchetiana*	Xiang CL, 287 (KUN)	Yunan, China	MF193532	MF193578
*Scutellaria sessilifolia*	Xiang CLi, 341 (KUN)	Chongqing, China	MF193533	MF193579
*Scutellaria sessilifolia*	Peng H et al., 117 (KUN)	Sichuan, China	MF193534	MF193580
*Scutellaria galericulata*	M-14212	Iran	MF193535	MF193581
*Scutellaria regeliana*	Jiang L, 149 (KUN)	Neimenggu, China	MF193536	MF193582
*Scutellaria dependens*	Anonymous, 565	Fujinomiya, Japan	MF193537	MF193583
*Scutellaria dependens*	Anonymous, 316	Fujinomiya, Japan	MF193538	MF193584
*Scutellaria barbata*	Xiang CL, 282 (KUN)	Beijing, China	MF193539	MF193585
*Scutellaria scordifolia*	Yu WT et al., 2822 (KUN)	Qinhai, China	MF193540	MF193586
*Scutellaria diffusa*	Wang ZH, s.n(KUN)	Germany	MF193541	MF193587
*Scutellaria kingiana*	Zhang JW et al., ZJW-3890 (KUN)	Xizang, China	MF193542	MF193588
*Scutellaria nuristanica*	M-32142	Iran	–	MF193589
*Scutellaria stocksii*	M-30348	Iran	MF193543	MF193590
*Scutellaria alpina*	Liao PC, s.n.	Europe alpine region	MF193544	MF193591
*Scutellaria nepetifolia*	TUH-27605 (THU)	Iran	MF193545	MF193592
*Scutellaria platystegia*	TUH-7697(THU)	Iran	MF193546	MF193593
*Scutellaria supina*	LiuB et al., CPG28095 (PE)	Xinjiang, China	MF193547	MF193594
*Holmskioldia sanguinea*	Anonymous, 209	Guandong, China	MF193548	MF193595
*Tinnea rhodesiana*	Gary Stafford, GIS-359 (KUN)	Pietermaritzburg, South Africa	MF193549	MF193596

For the molecular phylogenetic studies, the ingroup consisted of 45 samples (41 taxa) representing the two subgenera defined by Paton (1990a). *Holmskioldia sanguinea* Retz. and *Tinnea rhodesiana* S. Moore were used as the outgroup based on our previous studies (Li et al., 2016). Voucher information and GenBank numbers are provided in Table 1.

Total DNA was extracted from 0.3 g of silica-gel-dried leaves or from leaf fragments of herbarium specimens using a modified CTAB protocol by Soltis et al. (1991). The primer pair ITS4 and ITS5 (White et al., 1990) was used to amplify the entire nrITS region, and primers ETS-B from Beardsley & Olmstead (2002) and 18S-IGS from Baldwin & Markos (1998) were used to amplified the ETS region. The PCR reaction mixtures and program follow Chen et al. (2005).

Sequencing reactions were performed with the dideoxy chain termination method running on an ABI PRISM3730 automated sequencer. The same primers described above for PCR were used for the sequencing reactions. Sequences were assembled and edited with SeqMan (DNASTAR, Madison, Wisconsin, USA; Burland, 2000). Initial automated alignments of nrITS and ETS sequences were made using MEGA version7.0 (Kumar, Stecher & Tamura, 2016) and subsequently adjusted manually based on the similarity criterion in PhyDE v.0.997 (http://www.phyde.de/index.html). Gaps were treated as missing data. Finally, the matrices were concatenated in Phyutility v.2.2 (Smith & Dunn, 2008) and phylogenetic analyses conducted using Maximum likelihood (ML) and Bayesian inference (BI) methods. ML analyses were performed using RAxML HPC2 v.8.2.10 (Stamatakis, 2014), on the CIPRES Science Gateway v.3.3 (Miller, Pfeiffer & Schwartz, 2010). A partitioned model (-q) was selected, and 1,000 bootstrap iterations (-# — -N) were conducted, with other parameters using the default settings. BI analyses were implemented using the program MrBayes v.3.2.2 (Ronquist et al., 2012). The best-fit nucleotide substitution model (GTR +I +G) selected by Akaike information criterion (AIC) in Modeltest v.3.7 (Posada & Crandall, 1998). The Markov chain Monte Carlo (MCMC) algorithm was run for 10,000,000 generations with four chains, and trees were sampled every 100 generations. The first 25% of sampled generations were discarded as burnin, and a 50% majority-rule consensus tree was obtained using the remaining trees.

The electronic version of this article in Portable Document Format (PDF) will represent a published work according to the International Code of Nomenclature for algae, fungi, and plants (ICN), and hence the new name contained in the electronic version are effectively published under that Code from the electronic edition alone. Additionally, the new name contained in this work has been issued with identifiers by IPNIand will eventually be made available to the Global Name Index. The IPNI LSIDs can be resolved and the associated information viewed through any standard web browser by appending the LSID contained in this publication to the prefix “http://ipni.org/.” The online version of this work is archived and available from the following digital repositories: PeerJ, PubMed Central and CLOCKSS.

## Results

### Morphological studies

**Figure 1:**
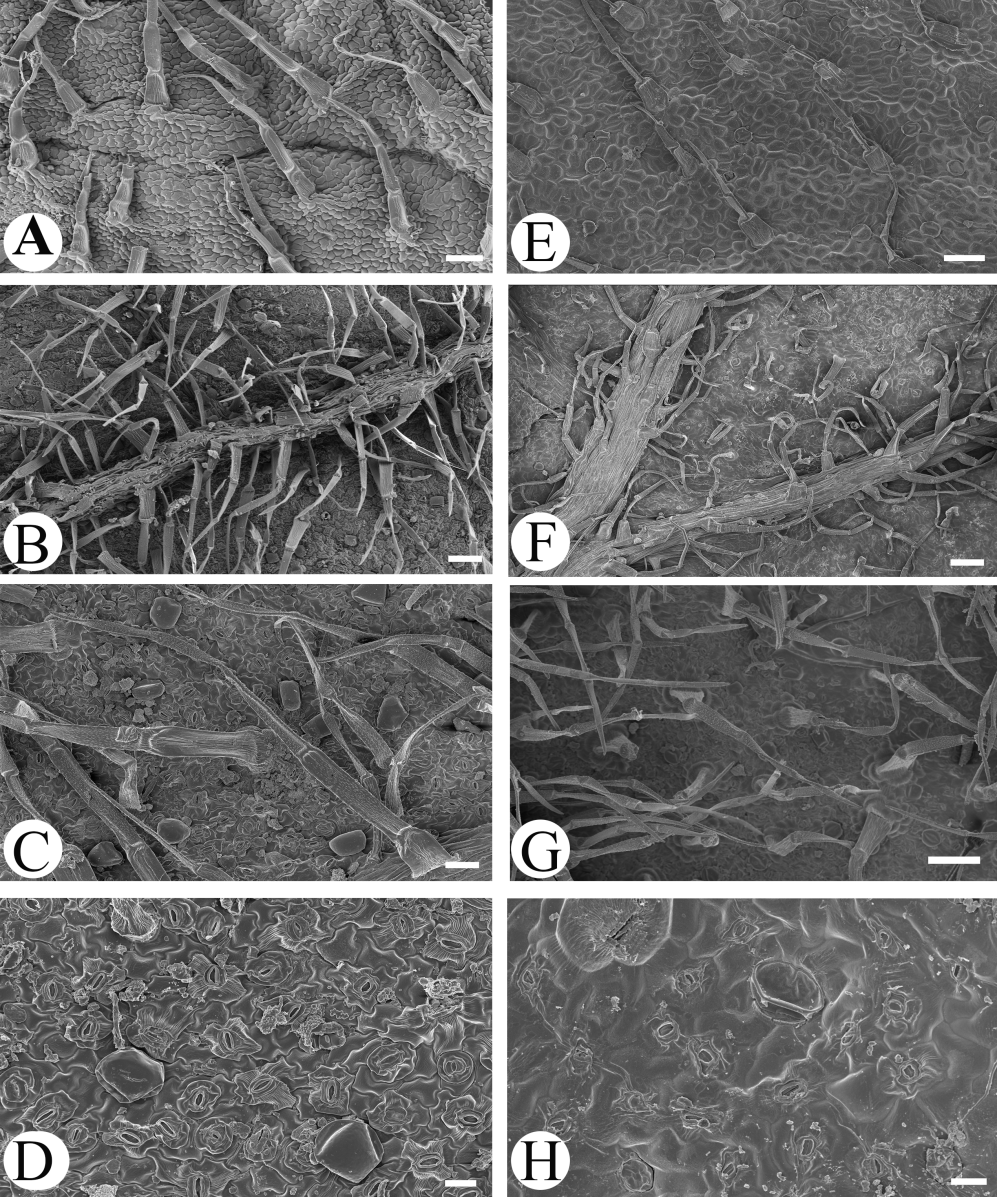
Scanning electron micrographs of leaf epidermis of *Scutellaria wuana* (A–D), *S. mairei* (E–H). (A) Non-glandular trichomes on adaxial surface in *S. wuana.* (I) Capitate glandular trichomes mixed with non-glandular on adaxial surface in *S. mairei.* (B, F) Non-glandular trichomes and capitate glandular trichomes distribute along the veins. (C, G) Non-glandular trichomes and capitate glandular trichomes on abaxial surface. (D, H) Stomata on abaxial surface. Scale bars = 100 μm (A, B, E, F, G), 40 μm (C), 20 μm (D, H).

**Figure 2:**
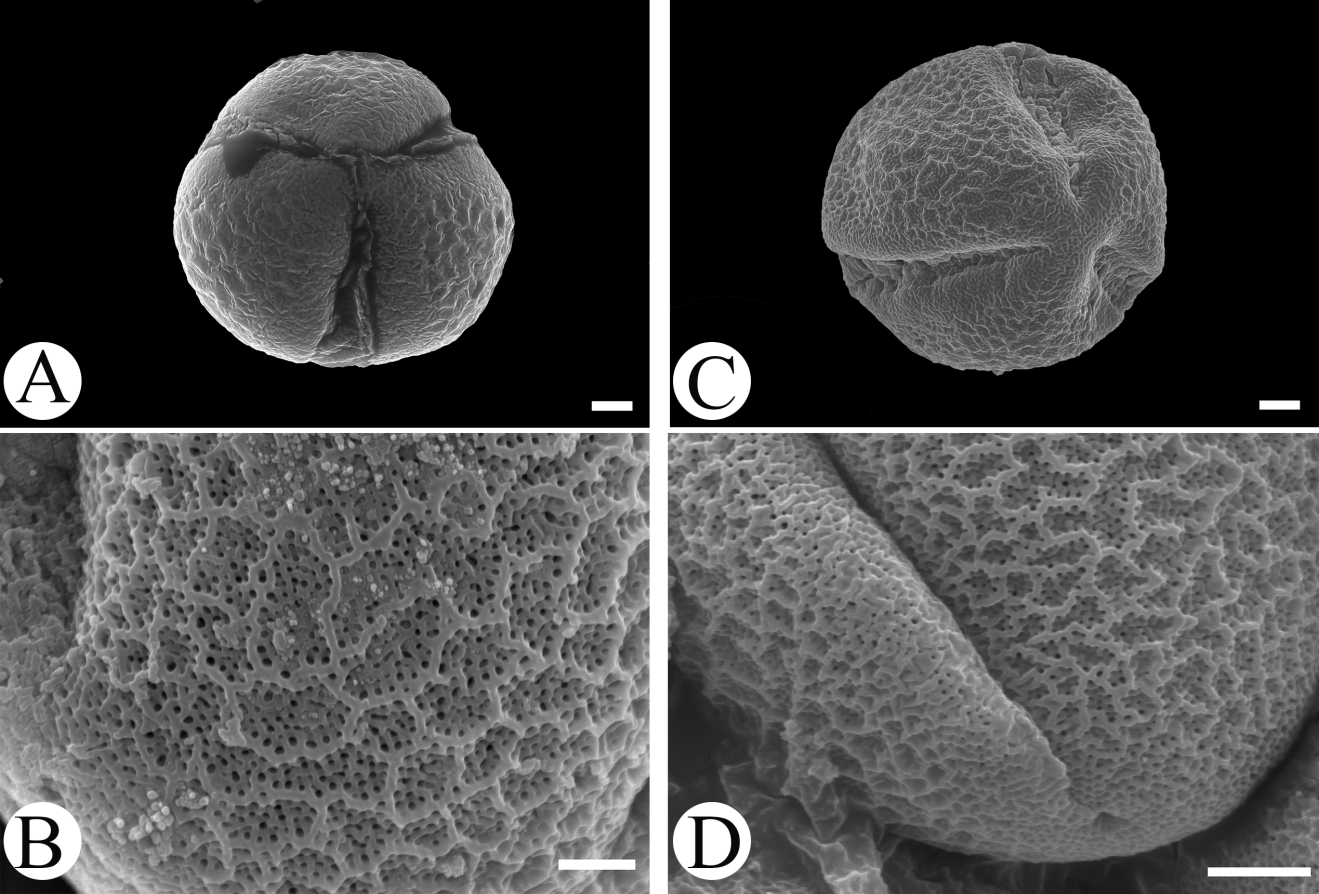
Scanning electron micrographs of pollen grains for *Scutellaria wuana* (A, B) and *S. mairei* (C, D). (A, C) Pollen grain (polar view), (B, D) exine surface. Scale bars = 2 μm (A–C), 1.5 μm (D).

Microfeatures of the leaf epidermis of the new species (Figs. 1A–1D) and *Scutellaria mairei* (Figs. 1E–1H) are shown in Fig. 1. Epidermal cells for both species were polygonal to slightly irregular in shape (Figs. 1A, 1E). Leaf surfaces were all hypostomatic, and anomocytic stomata were present in the abaxial epidermis (Figs. 1D, 1H). Glandular and non-glandular trichomes were found. The capitate glandular trichomes can only be found on the abaxial surface in *S. wuana* (Figs. 1C, 1D), but on both surfaces in *S. mairei* (Figs. 1E, 1G). The multicellular non-glandular trichomes were present on both surfaces (Figs. 1A, 1C, 1E, 1G), and densely distributed along the midrib and veins on the abaxial surface (Figs. 1B, 1F). The pollen grains of *S. wuana* (Figs. 2A–2B) and *S. mairei* (Figs. 2C–2D) are tricolpate, with a circular outline in polar view (Figs. 2A, 2C). The exine sculpturing in both species possess bireticulate perforations (Figs. 2B–2D).

### Molecular systematics

**Figure 3:**
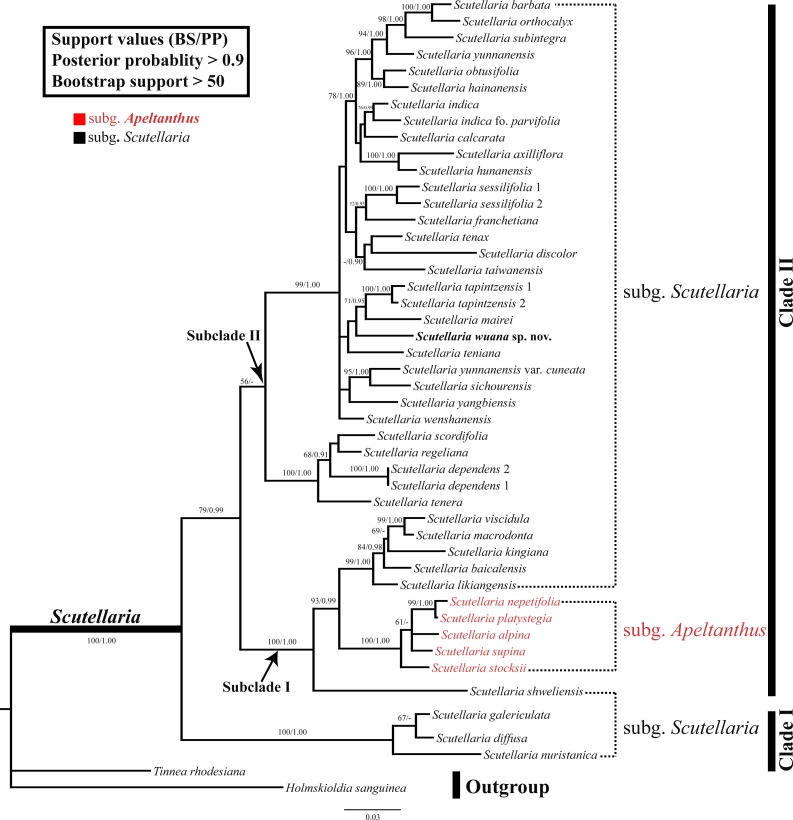
BI tree inferred from the combined dataset of nrITS and ETS sequences. With support values (BS/PP) indicated at branches. The outgroup and recognized clades summarized by the right bars.

The combined nrDNA dataset contained 1,110 positions, of which 332 characters were parsimony-informative characters. The topologies were congruent between the trees obtained in the ML and BI analyses, and thus only the BI topology tree is provided (Fig. 3). In both analyses, the monophyly of the *Scutellaria* is strongly supported (ML-BS = 100%, BI-PP = 1.00; all values follow this order hereafter) and consists of two main clades (Fig. 3). Clade I (100%, 1.00) comprises three species, *Scutellaria galericulata* L., *S. diffusa* Benth., and *S. nuristanica* Rech.f. Clade II (78%, 0.99) consists of the remaining species and two subclades can be identified. Subclade I (100%, 1.00) contains *S. shweliensis* W.W. Sm., *S. stocksii* Boiss., *S. alpina* L., *S. supina* L., *S. nepetifolia* Benth., *S. platystegia* Juz., *S*. *likiangensis* Diels, *S*. *baicalensis* Georgi, *S*. *kingiana* Prain, *S*. *viscidula* Bunge, and *S*. *macrodonta* Hand.-Mazz. It is noteworthy that within this subclade, five species from subgen. *Apeltanthus* formed a clade with moderate support values (65%, 0.91). Subclade II (57%, −) contains the remainder of *Scutellaria* and is shown as a polytomy. The new species we described here is a member of this subclade, and grouped with *S. teniana* Hand.-Mazz. *S. mairei*, and *S. tapintzensis* C.Y. Wu & H.W. Li., but with weak support.

### Taxonomic treatment

**Figure 4:**
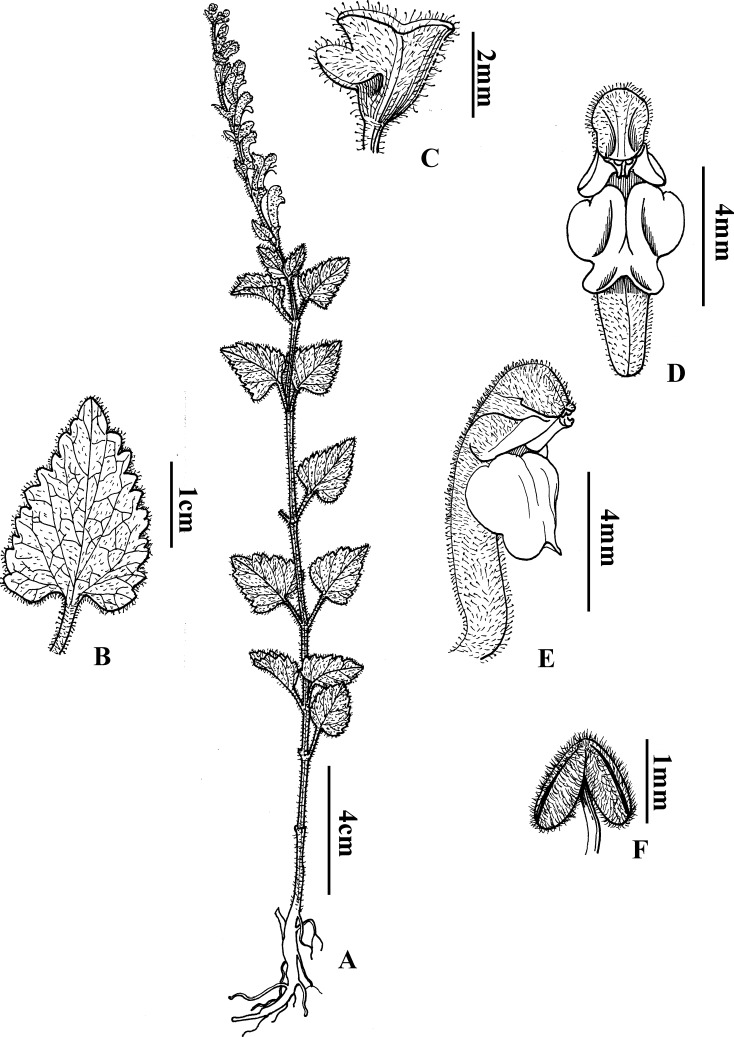
Diagnostic morphologcial features of *Scutellaria wuana* C. L. Xiang & F. Zhao (*C.L. Xiang 1200*) (Holotype). (A) Habit. (B) Leaf blade. (C) Calyx. (D) Flower in frontal view. (E) Flower in lateral view. (F) Anther. Drawing by Lin Wang.

**Figure 5:**
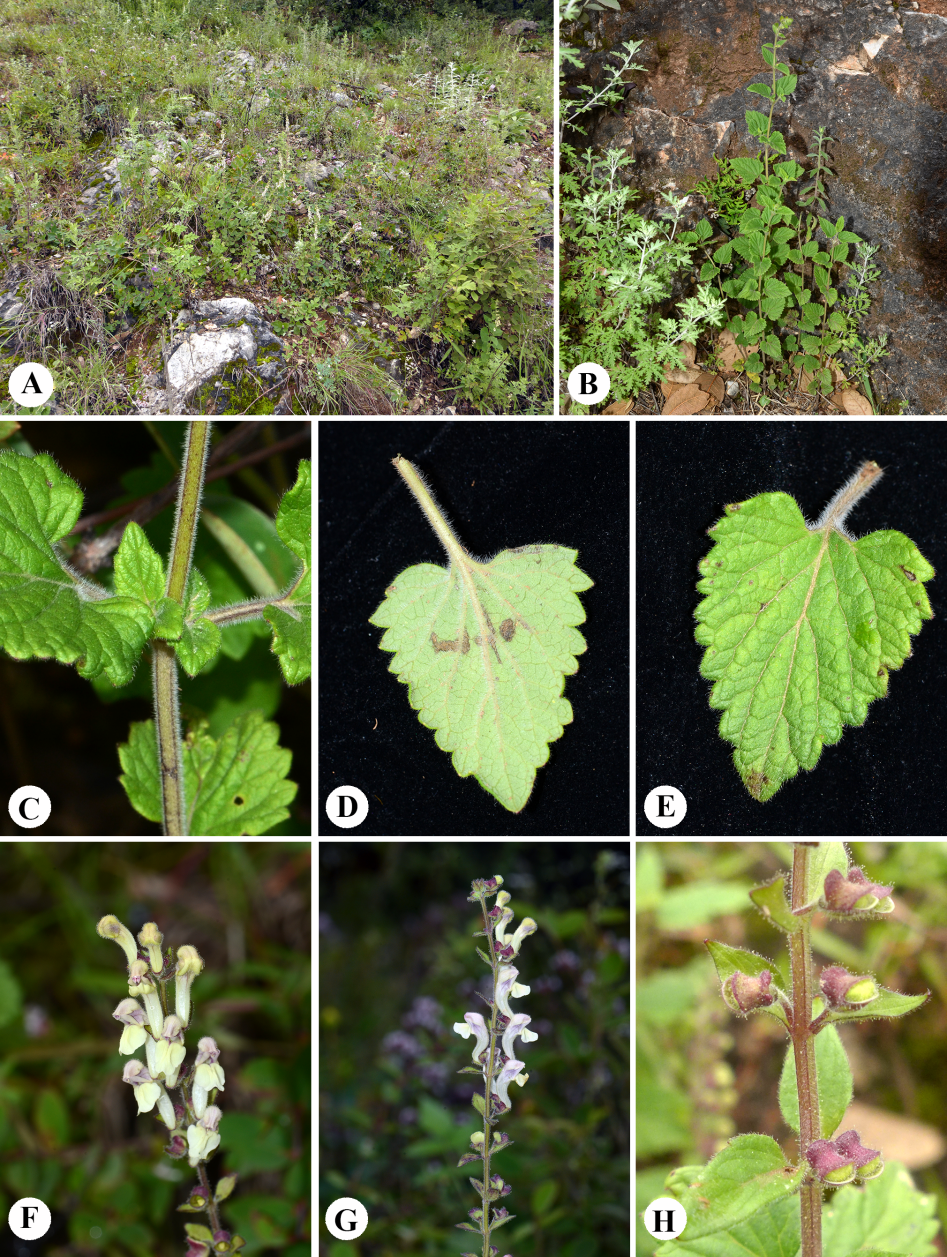
Photographs of *Scutellaria wuana* in the wild. (A) Plant habitat. (B) Habitat. (C) Stem. (D) Abaxial surface of leaf. (E) Adaxial surface of leaf. (F) Inflorescence in frontal view. (G) Inflorescence in lateral view. (H) Fruiting calyces. Photographs by CL Xiang.

***Scutellaria wuana* C.L. Xiang & F. Zhao, sp. nov. (Figs. 4 and 5)**

***Type***

CHINA. Sichuan Province: Muli County, Xiamaidi Village, in rock-crevice in dry and warm valley, elevation 3,080 m, 27°43′11.5″N, 101°14′07.4″E, 25 August 2015, *C.L. Xiang 1200* (Holotype KUN!, isotypes PE!, KUN!).

***Diagnosis***

*Scutellaria wuana* is similar to *S. mairei* H. Lév. (Léveillé, 1912) but differs by having an erect stem with white pubescent hairs (*vs.* suberect stem with gray to yellowish hirsute hairs in *S. mairei*), a leaf petiole 1–1.5 cm long (*vs*. 1.5–3 mm in *S. mairei*), a yellow corolla tube with a pink spot on the galea (*vs*. limp rose corolla in *S. mairei*), the median lobe of the lower lip trapeziform (*vs*. broadly ovate in *S. mairei*), and an arcuate corolla tube base (*vs.* slightly saccate in *S. mairei*).

***Description***

Perennial herb. Rhizome slender and densely fibrous. Stems 20–60 cm tall, erect, densely white pilose, glandular pubsecent on angles, apically few-branched. Leaves papery, lamina triangular to triangular-cordate, 15–38 mm long, 10–15 mm wide, base broadly cuneate to cordate, margin crenate-serrate, apex obtuse, adaxially sparsely pilose (Fig. 1A), abaxially densely white hirtellous; midrib and veins pubescent (Fig. 1B); petiole 1–1.5 cm. One sided racemes terminal or terminal in axillary branches, 8–14 cm long; bracts sessile, rhombic-ovate, 2–5 mm, leaf-like basally, margin glandular puberulent. Pedicel ca. 2.5 mm, densely white glandular puberulent. Calyx ca. 2 mm, elongate to 5 mm in fruit, glandular puberulent outside; scutellum ca. 1 mm, elongate to 3 mm in fruit. Corolla tube yellow with pink spot on galea, 12–15 mm, densely white glandular puberulent outside, glabrous inside; tube zigzag, ca. 1 cm long, base arcuate; throat ca. 6 mm wide; upper lip galeate, lower lip 3-lobed, median lobe trapeziform, apex emarginate, lateral lobes oblong-ovate, ca. 4 mm wide. Nutlets unseen. Fl. Jul–Aug.

***Distribution and Habitat***

**Figure 6:**
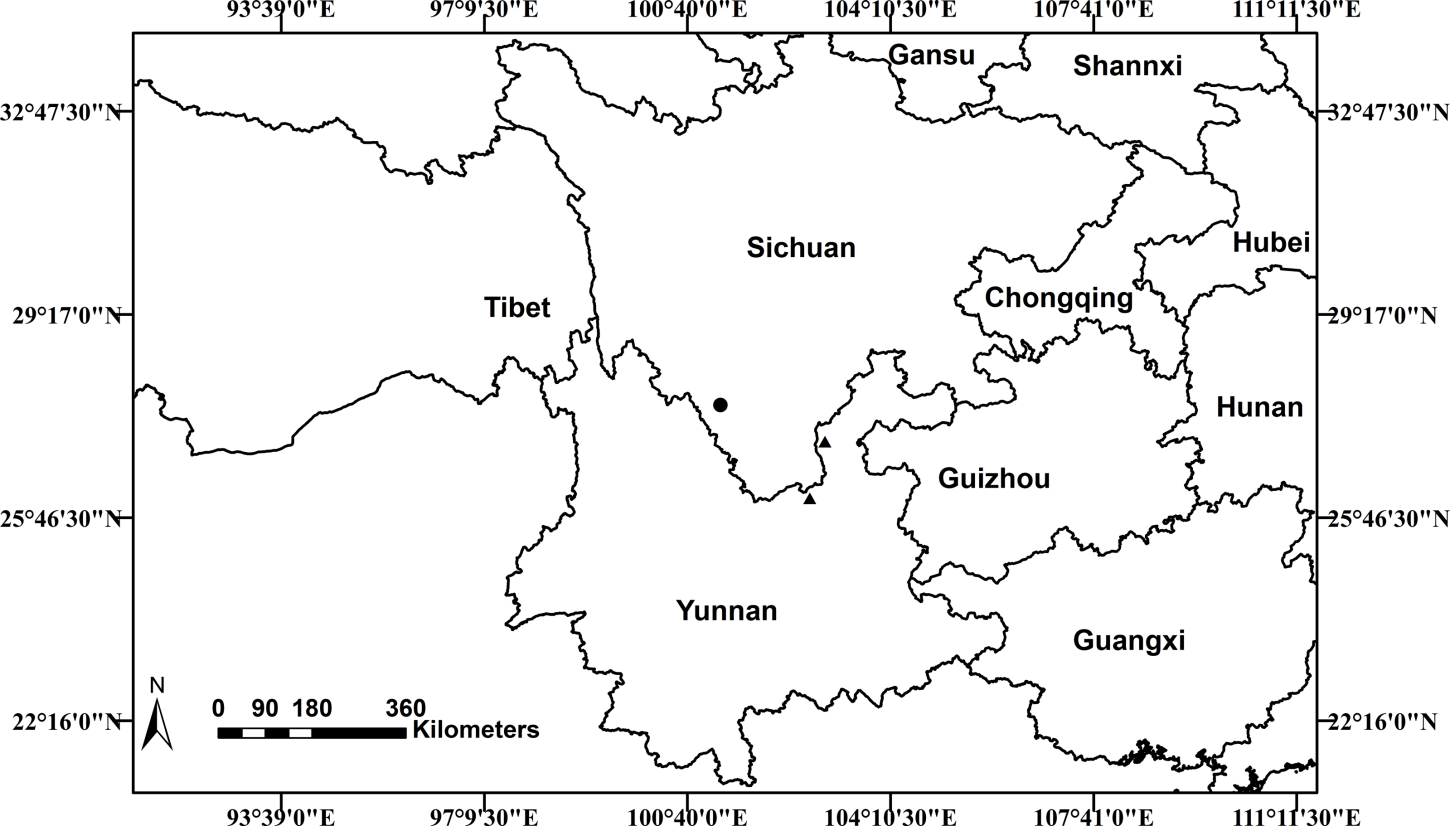
Distribution map of *Scutellaria wuana* (•) *S. mairei* (▴) in China.

*Scutellaria wuana* is currently only known from Muli County, Sichuan Province, in southwest China. Based on the information from our own collections and herbarium specimens, a distribution map of *S. wuana* (solid circle) and *S. mairei* (triangle) is shown in (Fig. 6). *Scutellaria wuana* is found in vegetation mainly composed of herbaceous and shrub by plants such as *Boenninghausenia albiflora* (Hook.) Meisn., *Campanula colorata* Wall., *Hypericum monogynum* L., *Origanum vulgare* L., *Salvia castanea* Diels and *Artemisia* sp., etc. More fieldwork is needed to further elucidate its habitat and distribution area.

***Etymology***

The epithet of the new species is named in honor of Professor Cheng-Yi Wu (Zheng-Yi Wu; 1916–2013), a prominent botanist from Kunming Institute of Botany, Chinese Academy of Sciences, to honor his great contributions to the knowledge of the family Lamiaceae in China and his significant work on the flora and vegetation of China for over 70 years.

***Additional specimens examined***

***Scutellaria wuana*** **C.L. Xiang & F. Zhao CHINA. Sichuan:** Muli Couunty, Xiamaidi Villlage, 26 July 2011, *E.D. Liu et al. 2902*. (KUN!) (Paratype).

***Scutellaria mairei*** **H. Lév. CHINA.** **Yunnan:** Qiaojia County, 1913, *E.E. Marie.* 510 (K!); 22 July 1909, *F. Ducloux 6438* (KUN!); 2 July 2004, *H*. *Wang et al. 03-1469* (KUN!); Luquan County, 4 August 2008, *H*. *Peng 9620* (KUN!).

## Discussion

The monophyly of the genus *Scutellaria* was confirmed in the present study (Fig. 3), as reported by previous molecular phylogenetic studies (Li et al., 2012; Li et al., 2016; Xiang et al., 2013; Chen et al., 2014; Chen et al., 2016). However, the subgenus *Scutellaria* defined by Paton (1990a) is non-monophyletic with members of the subgenus scattered in various clades. In addition, all the sections and series defined by Wu & Li (1977) and all groups defined by Li & Hedge (1994) are also not monophyletic. The subgenus *Apeltanthus* circumscribed by Paton (1990a) formed a small subclade and it is probably monophyletic (subclade ‘*Apeltanthus*’), but only five species were selected in this study, and future studies including more species from different regions are needed to test the monophyly of this subgenus. The taxonomic level and/or circumscription of the subgen. *Apeltanthus* should be reconsidered, because subclade *Apeltanthus* is embedded within subgenus *Scutellaria*. Future studies of morphological characters in concert with molecular data may provide better evidence for relationships among *Scutellaria* species, and could clarify the infrageneric classification of the genus.

As shown in (Fig. 3), *Scutellaria wuana* is a member of subgenus *Scutellaria*. Since *Scutellaria wuana* typically has a one-sided inflorescence that is composed of secund flowers and leaf-like bracts, it should be placed in the sect. *Scutellaria*. However, because all the sections are not monophyletic based on our molecular studies, it is unreasonable to place the new species based on this artificial infrageneric classification. Because *S. wuana* has a zigzag corolla tube and the leaf margin is denticulate to dentate, we speculate that it has a close relationship with the group defined by Li & Hedge (1994) that includes *S. mairei*, *S. tenax* W.W. Sm., *S. teniana*, and *S. tapintzenisis*. After careful comparison of the specimens of those species in combination with the results from our molecular phylogenetic study, we conclude that *S. wuana* is most closely related to *S. mairei*. Morphologically, both species are perennial herbs with slender rhizomes, triangular leaves with denticulate to dentate margins, and a zigzag corolla tube. However, *S. wuana* can be readily distinguished from *S. mariei* by several characters. Additionally, the distribution and habitat are different in the two species. *Scutellaria wuana* is currently only known from Muli County, Sichuan Province, and grow in rock-crevices within a xeric valley, while *S. mairei* is only found on dry limestone mountains in northeast Yunnan.The external morphology of *S. wuana* is more or less comparable with S. *tenax*; both species are perennial herbs and have triangular leaves and a zigzag corolla tube. However, there are several characters that distinguish *S. wuana* from *S. tenax*. Detailed comparisons of the morphology, habitat, and distribution of *S. wuana*, *S. tenax and S. mairei* are given in Table 2.

**Table 2:** Comparison of morphology, habitat, and distribution between *Scutellaria wuana*, *S. mairei.* and *S. tenax.*

Characters	*S. mairei*	*S. wuana*	*S. tenax*
Stem	suberect, 16–22 cm tall, with dense yellow long-hirsute hairs	erect, 20–80 cm,tall, densely with white pubescence	erect, ca 60 cm tall, with white pubescence
Lamina	papery, petiole 1.5–3 mm	papery, petiole 1–1.5 cm	membranous, petiole 0.5–1.8 cm
Corolla color	tube white, corolla limb rose	tube yellow, with pink spot in galea	tube base yellow, galea blue
Corolla tube	zigzag, base slightly dilated	zigzag, base arcuate	zigzag, base bent
Middle lobe of lower lip shape	broadly ovate	trapeziform	broadly oblong-ovate
Habitat	dry limestone mountains	valley	streamside, grasslands, thickets, forests
Distribution area	northeast Yunnan	Muli, Southwest Sichuan	North Yunnan & West Sichuan

Relationships between *Scutellaria wuana* and its allies (i.e., *S. teniana*, *S. mairei* and *S. tapintzensis*) were not fully resolved. These species formed a clade in our analyses (Fig. 3), but received meager support values. Part of the reason is that only two nuclear DNA regions were used for analyses in this study, and we predict that the use of more chloroplast DNA markers and broad phylogenomic sampling will improve the resolution of the phylogeny. Currently, it is difficult to resolve the species relationships for such a large genus. To this end a multi-disciplinary method including molecular systematics, phylogenomics, morphological anatomy, and taxonomy is necessary for future studies.

##  Supplemental Information

10.7717/peerj.3624/supp-1 Supplemental Information 1 Aligned ITS sequences dataset

10.7717/peerj.3624/supp-2 Supplemental Information 2 Aligned ETS sequences dataset

10.7717/peerj.3624/supp-3 Supplemental Information 3 Voucher information for phylogenetic analyses and GenBank accession numbers

## Additional Information and Declarations

### DNA Deposition

The following information was supplied regarding the deposition of DNA sequences: Species, ITS, ETS: *Scutellaria discolor* MF193504 MF193550
*Scutellaria hainanensis* MF193505 MF193551
*Scutellaria yunnanensis* var. *yunnanensis* MF193506 MF193552
*Scutellaria yunnanensis* var. *cuneata* MF193507 MF193553
*Scutellaria obtusifolia* MF193508 MF193554
*Scutellaria sichourensis* MF193509 MF193555
*Scutellaria wenshanensis* MF193510 MF193556
*Scutellaria yangbiense* MF193511 MF193557
*Scutellaria calcarata* MF193512 MF193558
*Scutellaria indica* MF193513 MF193559
*Scutellaria indica* fo. *parvifolia* MF193514 MF193560
*Scutellaria taiwanensis* MF193515 MF193561
*Scutellaria mairei* MF193516 MF193562
*Scutellaria tenax* MF193517 MF193563
*Scutellaria tapintzeensis*_1 MF193518 MF193564
*Scutellaria tapintzeensis*_2 MF193519 MF193565
*Scutellaria teniana* MF193520 MF193566
*Scutellaria wuana* sp. nov. MF193521 MF193567
*Scutellaria tenera* MF193522 MF193568
*Scutellaria macrodonta* MF193523 MF193569
*Scutellaria likiangensis* MF193524 MF193570
*Scutellaria baicalensis* MF193525 MF193571
*Scutellaria viscidula* MF193526 MF193572
*Scutellaria orthocalyx* MF193527 MF193573
*Scutellaria subintegra* MF193528 MF193574
*Scutellaria axilliflora* MF193529 MF193575
*Scutellaria shweliensis* MF193530 MF193576
*Scutellaria hunanensis* MF193531 MF193577
*Scutellaria franchetiana* MF193532 MF193578
*Scutellaria sessilifolia* MF193533 MF193579
*Scutellaria sessilifolia* MF193534 MF193580
*Scutellaria galericulata* MF193535 MF193581
*Scutellaria regeliana* MF193536 MF193582
*Scutellaria dependens* MF193537 MF193583
*Scutellaria dependens* MF193538 MF193584
*Scutellaria barbata* MF193539 MF193585
*Scutellaria scordifolia* MF193540 MF193586
*Scutellaria diffusa* MF193541 MF193587
*Scutellaria kingiana* MF193542 MF193588
*Scutellaria nuristanica* - MF193589
*Scutellaria stocksii* MF193543 MF193590
*Scutellaria alpina* MF193544 MF193591
*Scutellaria nepetifolia* MF193545 MF193592
*Scutellaria platystegia* MF193546 MF193593
*Scutellaria supina* MF193547 MF193594
*Holmskioldia sanguinea* MF193548 MF193595
*Tinnea rhodesiana* MF193549 MF193596.

### Data Availability

The following information was supplied regarding data availability: Accession numbers can be found in Table 1. Data for sequences can also be found in the Supplemental Information.

### New Species Registration

The following information was supplied regarding the registration of a newly described species: *Scutellaria wuana* LSID: 77164113-1.
