# Supplementary material for: A new species of Scutellaria (Scutellarioideae, Lamiaceae) from Sichuan Province in southwest China
Source: PeerJ. 2017 Aug 8;5:e3624. doi: 10.7717/peerj.3624 (PMC5550825; doi:10.7717/peerj.3624)
Supplement: Supplemental Information 3 [file peerj-05-3624-s003.docx]

**Voucher information for phylogenetic analyses and GenBank accession numbers (see also Table 1).**

| **Taxon** | **Voucher/Herbarium barcode** | **Location** | **Genbank accession number** | |
| --- | --- | --- | --- | --- |
|  |  |  | **ITS** | **ETS** |
| *Scutellaria discolor* | Xiang CL et al., 438 (KUN) | Yunnan, China | MF193504 | MF193550 |
| *Scutellaria hainanensis* | Jiang L et al., 398 (KUN) | Hainan, China | MF193505 | MF193551 |
| *Scutellaria yunnanensis* var. *yunnanensis* | Liu Ed et al., 3037 (KUN) | Yunnan, China | MF193506 | MF193552 |
| *Scutellaria yunnanensis* var. *cuneata* | Xiang CL et al., 574 (KUN) | Yunnan, China | MF193507 | MF193553 |
| *Scutellaria obtusifolia* | Chen YP et al., EM202 (KUN) | Sichuan, China | MF193508 | MF193554 |
| *Scutellaria sichourensis* | Xiang CL et al., 566 (KUN) | Yunnan, China | MF193509 | MF193555 |
| *Scutellaria wenshanensis* | Zhao F et al., 008 (KUN) | Yunnan, China | MF193510 | MF193556 |
| *Scutellaria yangbiense* | Liu ED et al., 2238 (KUN) | Yunnan, China | MF193511 | MF193557 |
| *Scutellaria calcarata* | Shui YM et al., Z-03343396 (KUN) | Yunnan, China | MF193512 | MF193558 |
| *Scutellaria indica* | Peng H, s.n (KUN) | Hongkong, China | MF193513 | MF193559 |
| *Scutellaria indica* fo. *parvifolia* | Anonymous, 554 (KUN) | Shimoda , Japan | MF193514 | MF193560 |
| *Scutellaria taiwanensis* | Liao PC, s.n. (KUN) | Taiwan, China | MF193515 | MF193561 |
| *Scutellaria mairei* | Shui YM et al., 66205 (KUN) | Yunnan, China | MF193516 | MF193562 |
| *Scutellaria tenax* | Peng H et al., 2012-017 (KUN) | Guizhou, China | MF193517 | MF193563 |
| *Scutellaria tapintzeensis*_1 | Cai J et al., 15cs11358 (KUN) | Yunnan, China | MF193518 | MF193564 |
| *Scutellaria tapintzeensis*_2 | Cai J et al., 15cs11371 (KUN) | Yunnan, China | MF193519 | MF193565 |
| *Scutellaria teniana* | Xiang CL et al., 288 (KUN) | Yunnan, China | MF193520 | MF193566 |
| *Scutellaria wuana* sp. nov. | Xiang CL et al., 1200 (KUN) | Sichuan, China | MF193521 | MF193567 |
| *Scutellaria tenera* | Chen YP et al., EM187 (KUN) | Jiangxi, China | MF193522 | MF193568 |
| *Scutellaria macrodonta* | Zhao F et al., 2015-006 (KUN) | Beijing, China | MF193523 | MF193569 |
| *Scutellaria likiangensis* | Xiang CL et al., 373 (KUN) | Yunnan, China | MF193524 | MF193570 |
| *Scutellaria baicalensis* | Li DZ et al., 0513 (KUN) | Liaoning, China | MF193525 | MF193571 |
| *Scutellaria viscidula* | Zhao F, 2015-009 (KUN) | Hebei, China | MF193526 | MF193572 |
| *Scutellaria orthocalyx* | Xiang CL, 185 (KUN) | Yunnan, China | MF193527 | MF193573 |
| Table 1 (continued) |  |  |  |  |
| *Scutellaria subintegra* | Chen YP, EM223 (KUN) | Guangxi, China | MF193528 | MF193574 |
| *Scutellaria axilliflora* | Hu GX, H144 (KUN) | Fujian, China | MF193529 | MF193575 |
| *Scutellaria shweliensis* | Zhao F et al., ZF0068 (KUN) | Yunnan, China | MF193530 | MF193576 |
| *Scutellaria hunanensis* | Hu GX, H96 (KUN) | Hunan, China | MF193531 | MF193577 |
| *Scutellaria franchetiana* | Xiang CL, 287 (KUN) | Yunan, China | MF193532 | MF193578 |
| *Scutellaria sessilifolia* | Xiang CLi, 341 (KUN) | Chongqing, China | MF193533 | MF193579 |
| *Scutellaria sessilifolia* | Peng H et al., 117 (KUN) | Sichuan, China | MF193534 | MF193580 |
| *Scutellaria galericulata* | M-14212 | Iran | MF193535 | MF193581 |
| *Scutellaria regeliana* | Jiang L, 149 (KUN) | Neimenggu, China | MF193536 | MF193582 |
| *Scutellaria dependens* | Anonymous, 565 | Fujinomiya, Japan | MF193537 | MF193583 |
| *Scutellaria dependens* | Anonymous, 316 | Fujinomiya, Japan | MF193538 | MF193584 |
| *Scutellaria barbata* | Xiang CL, 282 (KUN) | Beijing, China | MF193539 | MF193585 |
| *Scutellaria scordifolia* | Yu WT et al., 2822 (KUN) | Qinhai, China | MF193540 | MF193586 |
| *Scutellaria diffusa* | Wang ZH, s.n (KUN) | Germany | MF193541 | MF193587 |
| *Scutellaria kingiana* | Zhang JW et al., ZJW-3890 (KUN) | Xizang, China | MF193542 | MF193588 |
| *Scutellaria nuristanica* | M-32142 | Iran | - | MF193589 |
| *Scutellaria stocksii* | M-30348 | Iran | MF193543 | MF193590 |
| *Scutellaria alpina* | Liao PC, s. n. | Europe alpine region | MF193544 | MF193591 |
| *Scutellaria nepetifolia* | TUH-27605 (THU) | Iran | MF193545 | MF193592 |
| *Scutellaria platystegia* | TUH-7697 (THU) | Iran | MF193546 | MF193593 |
| *Scutellaria supina* | Liu B et al., CPG28095 (PE) | Xinjiang, China | MF193547 | MF193594 |
| *Holmskioldia sanguinea* | Anonymous, 209 | Guandong, China | MF193548 | MF193595 |
| *Tinnea rhodesiana* | Gary Stafford, GIS-359 (KUN) | Pietermaritzburg, South Africa | MF193549 | MF193596 |
